# Registration of 2D C-Arm and 3D CT Images for a C-Arm Image-Assisted Navigation System for Spinal Surgery

**DOI:** 10.1155/2015/478062

**Published:** 2015-05-28

**Authors:** Chih-Ju Chang, Geng-Li Lin, Alex Tse, Hong-Yu Chu, Ching-Shiow Tseng

**Affiliations:** ^1^Department of Neurosurgery, Cathay General Hospital, Taipei City 10630, Taiwan; ^2^Department of Medicine, School of Medicine, Fu Jen Catholic University, New Taipei City 24205, Taiwan; ^3^Department of Mechanical Engineering, National Central University, Taoyuan County 32001, Taiwan

## Abstract

C-Arm image-assisted surgical navigation system has been broadly applied to spinal surgery. However, accurate path planning on the C-Arm AP-view image is difficult. This research studies 2D-3D image registration methods to obtain the optimum transformation matrix between C-Arm and CT image frames. Through the transformation matrix, the surgical path planned on preoperative CT images can be transformed and displayed on the C-Arm images for surgical guidance. The positions of surgical instruments will also be displayed on both CT and C-Arm in the real time. Five similarity measure methods of 2D-3D image registration including Normalized Cross-Correlation, Gradient Correlation, Pattern Intensity, Gradient Difference Correlation, and Mutual Information combined with three optimization methods including Powell's method, Downhill simplex algorithm, and genetic algorithm are applied to evaluate their performance in converge range, efficiency, and accuracy. Experimental results show that the combination of Normalized Cross-Correlation measure method with Downhill simplex algorithm obtains maximum correlation and similarity in C-Arm and Digital Reconstructed Radiograph (DRR) images. Spine saw bones are used in the experiment to evaluate 2D-3D image registration accuracy. The average error in displacement is 0.22 mm. The success rate is approximately 90% and average registration time takes 16 seconds.

## 1. Introduction

Conventionally, spinal surgery, especially minimally invasive spinal surgery, usually requires taking numerous C-Arm images to confirm that the positioning of surgical instruments is correct and safe, which leads to medical persons' high risk of radiation exposure [1]. C-Arm image-assisted surgical navigation system has been broadly applied to orthopedic surgery because C-Arm machine is commonly available for orthopedic surgery and registration between C-Arm images and the patient is automatic. Moreover, C-Arm image-assisted surgical navigation system needs only two C-Arm images taken in different angles to determine spatial target positions, which significantly reduces X-ray exposure dosage [2–5]. In recent years, using an image-assisted navigation system for spinal surgery has become a trend [3, 6, 7].

However, 2D C-Arm images lack 3D spatial information. Accurate path planning on the C-Arm AP-view image is difficult [6]. On the contrary, 3D CT images provide 3D anatomic information, which enables easy and safe path planning for spinal surgery. Therefore, path planning on CT images and guidance of surgical tools by C-Arm images are a good idea to integrate their advantages if C-Arm and CT images are registered accurately. This research evaluates the performance of several 2D-3D image registration methods to obtain the optimum transformation matrix between C-Arm and CT image frames and thus surgical paths planned on the CT images can be mapped onto the C-Arm images.

Among the known 2D-3D image registration methods, Markelj et al. [6] divided the existing rigid registration methods for 2D and 3D medical images into three types according to the data volume of image features, which are feature-based [8–10], gradient-based [2, 11], and intensity-based [1, 12–14]. Also, based on the image dimension and spatial connection, there are three registration methods for 2D C-Arm and 3D CT images: (1) the projection algorithm, which transforms a 3D image into 2D space for 2D-2D registration; (2) the back-projection algorithm; and (3) the 3D reconstruction algorithm, which transforms a 2D image into 3D space for 3D-3D registration [6]. By maximizing the similarity of the image contour, image gradient, or image gray scale, the registration result can coordinate the spatial locations of corresponding points on the two images.

2D-3D image registration aims to complete an accurate registration process within a short time, in order to improve the practicability in clinical operations. The accuracy of feature-based registration directly depends on the accuracy of segmentation, and it is therefore difficult to perform fully automatically. Gradient-based registration usually calculates complex and difficult convergences, while intensity-based registration operates the pixel intensity directly, without segmenting the target image to seek a corresponding feature point. This study evaluated the accuracy and time consumption of various methods and proposed the optimum 2D-3D image registration method for rapid and accurate registration of CT and C-Arm images. The aim of this research is to enable the self-developed C-Arm image-assisted navigation system to be practicable to minimally invasive spinal surgery.

## 2. Material and Methods

### 2.1. The C-Arm Image-Assisted Surgical Navigation System

**Figure 1:**
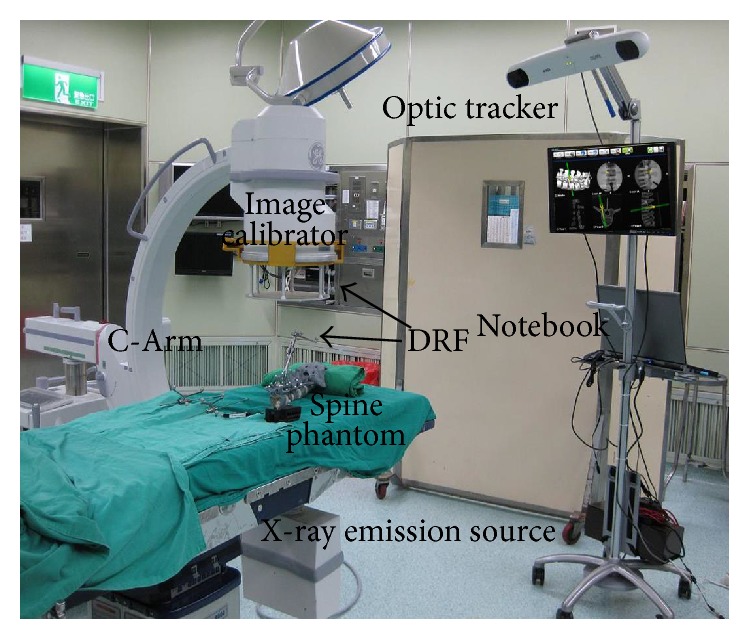
The self-developed C-Arm image-assisted surgical navigation system.

**Figure 2:**
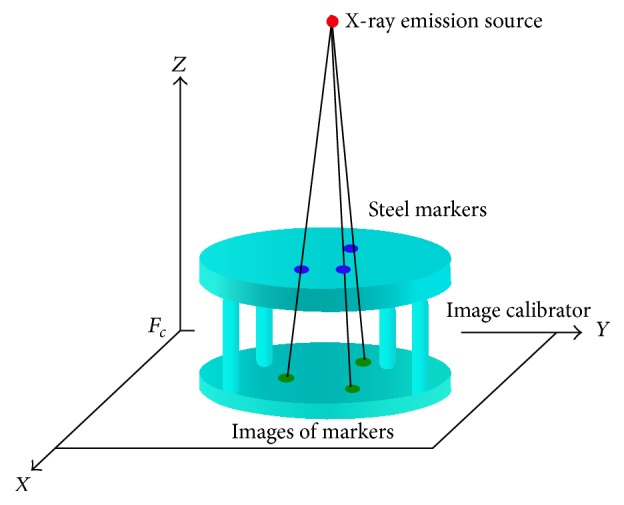
The X-ray projection model.

Figure 1 shows the self-developed C-Arm image-assisted surgical navigation system, which integrates Polaris Spectra passive optic tracker (Northern Digital Inc.) and a notebook with Intel CPU and an extra monitor to provide real-time display of navigation status. The optic tracker detects the positions and orientations of surgical tools, the spine, and the image calibrator attached on the C-Arm through the Dynamic Reference Frames (DRF). The double-deck image calibrator with feature markers (steel balls of different sizes) on up-deck and down-deck is designed for correction of X-ray image distortion and determination of the spatial position of X-ray emission source at the time of image taken.  Figure 2 illustrates the X-ray projection model defined by the position of the X-ray emission source and C-Arm image plane. The position of the X-ray emission source can be determined by finding the intersection of the projection lines passing though the up-deck steel makers of the image calibrator and their corresponding projection images.

**Figure 3:**
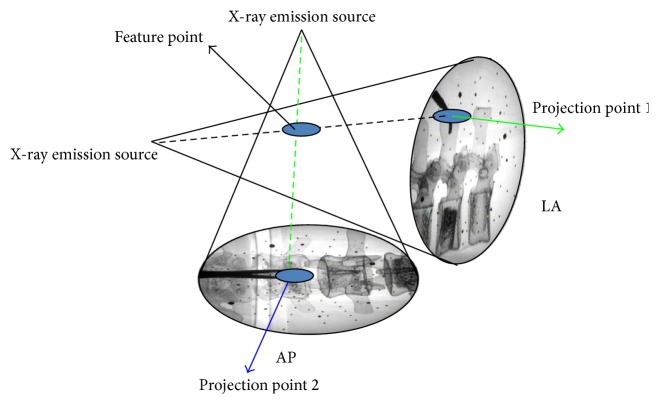
The spatial position of the feature point determined by its projection points.

Figure 3 shows an example of the C-Arm AP- and LA-view images. Ideally, C-Arm image-assisted surgical navigation system uses AP- and LA-view images to calculate the spatial location of the target point. The spatial position of any feature (target) point of the spine can be calculated by finding the intersection of the X-ray projection lines passing through the projection point of the feature point on each of the two C-Arm images. Further, the surgeon may plan a surgical path by selecting the projection points of the start point and end point of the path on each of the two C-Arm images. The navigation system will automatically calculate the spatial position and orientation of the surgical path. Under the real-time positioning guidance of the navigation system, the surgeon will be able to move surgical instruments tracked by the optic tracker to the planned surgical path. Since only two C-Arm images are needed for surgical planning and guidance, the risk of radiation exposure is reduced significantly compared to that of conventional surgery. Moreover, the surgeon will have more confidence in positioning surgical instruments and pedicle screws into the pedicle, and thus surgical quality can be improved [3].

Since C-Arm images are projective and with lack of spatial position information, it is difficult to plan surgical paths on C-Arm images. Instead, path planning in 3D CT reconstructed model is easy and accurate. Therefore, it is recommended to do path planning on 3D CT model and then transform the planned path onto the C-Arm images. This enables easy and safe path planning on the CT images and guidance of surgical tools by C-Arm images.

### 2.2. 2D C-Arm and 3D CT Registration

**Figure 4:**
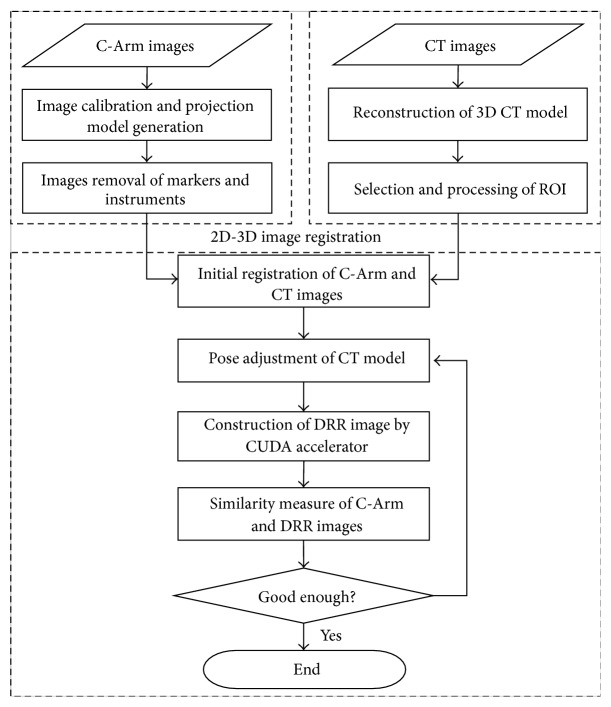
The procedure of 2D C-Arm and 3D CT images registration.

In order to transform the surgical path planned on CT images into the C-Arm images, 2D C-Arm and 3D CT registration is needed. The registration is to iteratively position the 3D CT model so that its Digital Reconstructed Radiograph (DRR) images and the C-Arm AP- and LA-view images have the highest image similarity.  Figure 4 shows the flowchart of the registration procedure including the following: (1) reconstructing 3D CT model; (2) calibrating C-Arm images and generating X-ray projection model of the C-Arm; (3) initial registration of C-Arm and CT images; (4) using CUDA to accelerate the DRR (Digital Reconstructed Radiograph) image construction; (5) calculating the similarity of C-Arm and DRR images and performing the optimization approach of registration.

**Figure 5:**
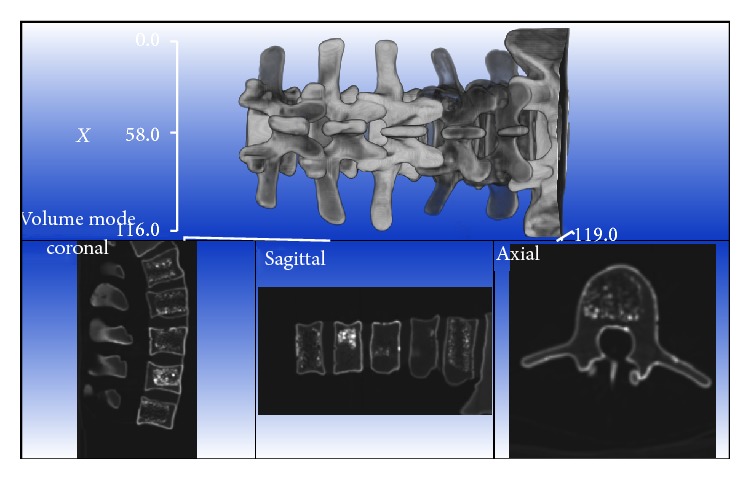
A reconstructed 3D CT spine model with axial, coronal, and sagittal views.

As shown in Figure 5, the 3D CT spine model is reconstructed by using marching cube algorithm [15]. The axial-, sagittal-, and coronal-view images are also generated for surgical path planning. The C-Arm AP- and LA-view images are taken, captured, and calibrated during the operation and the C-Arm X-ray projection model is constructed by the X-ray emission source and image plane, and the spatial geometry of the images is constructed using the biplanar method as shown in Figure 3. Three corresponding feature points on the 3D CT model and 2D C-Arm images are selected and used for initial registration between the CT model and the C-Arm images. Then, accurate registration is carried out by optimizing the similarity of the DRR and C-Arm images. This study used the intensity-based method for the 2D-3D image registration, which included the following three steps: (1) generating the DRR image according to the current pose of the CT model and the region of interest to save computing time; (2) measuring the similarity between the C-Arm and DRR images; (3) using the optimization approach to adjust the pose of the CT model iteratively, in order to obtain the optimum similarity of the C-Arm and DRR images; and (4) determining the transformation matrix between the CT and C-Arm image frames.

### 2.3. The Digital Reconstructed Radiograph (DRR)

The image gray level is positive proportional to the logarithm of received X-ray intensity. According to X-ray principle, the X-ray intensity projected onto an image plane can be calculated by

(1) $$\begin{array}{c} I\left(\;u,v\;\right)=I_{0}\exp\left(\;-\overset{}{\underset{r\left(u,v\right)}{\int }}\mu \left(\;x,y,z\;\right)dr\;\right), \end{array}$$

where *I*_0_ is the initial X-ray intensity, *I*(*u*, *v*) is the X-ray intensity received at position (*u*, *v*) of the C-Arm image plane, and *μ*(*x*, *y*, *z*, *E*_eff_) is the X-ray attenuation coefficient of the tissue at the position (*x*, *y*, *z*).

**Figure 6:**
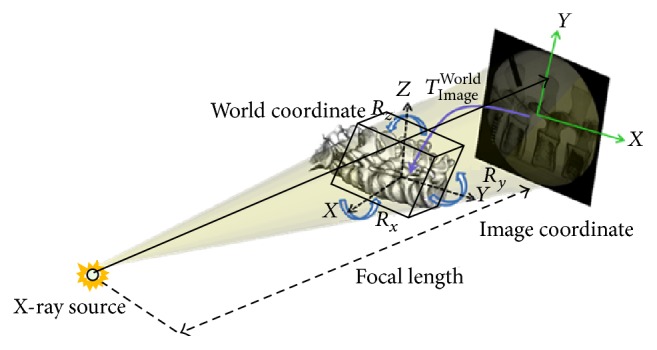
The ray-tracing projection model of DRR image.

For a voxel of CT images, its attenuation coefficient is positively related to its CT number or Hounsfield Units (HU). Therefore, the grey level of the DRR image pixel is determined based on the summation of CT numbers of the CT voxels passing through by the X-ray. In this study, ray casting method is selected to generate DRR images. The ray is determined by the X-ray emission source and a pixel of the C-Arm X-ray image plane. Also the ray has to pass through the ROI bounding box of the 3D CT spine model to save computer memory and computing time as shown in Figure 6. The accumulation of Hounsfield Units of all voxels passing through by the X-ray has linear relation with the DRR image grey level and is assigned to the range of 0~255.

The generation of a DRR image costs a lot of time, and thus the related optimization process of registration is time consuming, too. To accelerate the DRR reconstruction process, the parallel program development environment of Nvidia CUDA (GTX570 with 480 CUDA cores) is applied so that the projecting pose of CT spine model can be modified effectively to optimize the similarity of the C-Arm and DRR images rapidly, so as to enhance clinic practicability [16, 17]. An example to test the performance of the CUDA accelerator in generating DRR image from a set of 200 CT images has been done. The resolution of each CT image has a dimension of 512 × 512 pixels, while the DRR image size is set to be 470 × 470 pixels. The computing time of using NVIDIA GTX570 CUDA accelerator is 0.051 seconds while that of only using Intel CPU@2.4 GHz is 106.7 seconds. The performance of CUDA accelerator is significant.

**Figure 7:**
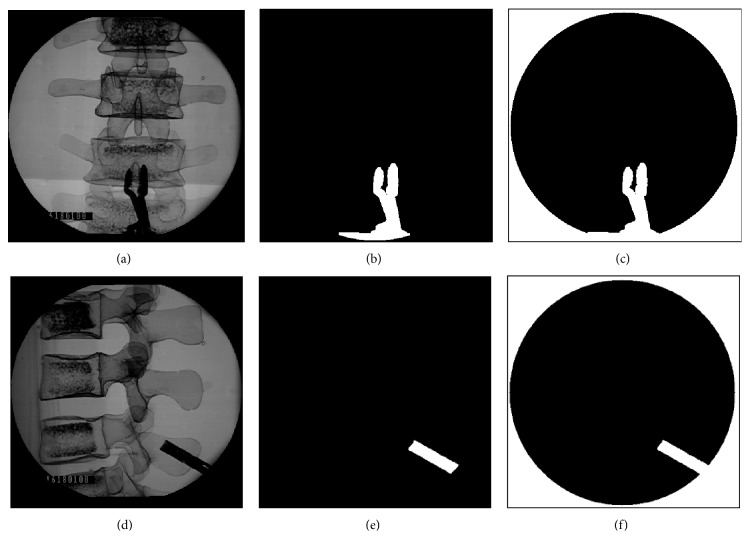
(a) Original AP-view image. (b) Segmentation of the instrument. (c) The mask for AP-view DRR image. (d) Original LA-view image. (e) Segmentation of the instrument. (f) The mask for LA-view DRR image.

**Figure 8:**
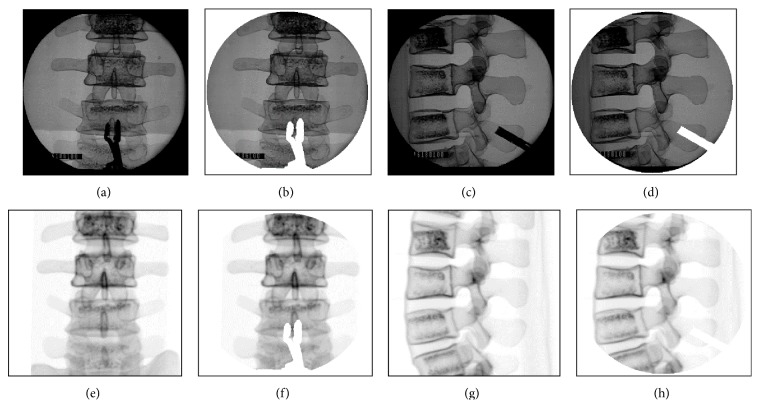
(a) Original AP-view image. (b) Effective AP-view image. (c) Original LA-view image. (d) Effective LA-view image. (e) Original AP-view DRR image. (f) Effective AP-view DRR image. (g) Original LA-view DRR image. (h) Effective LA-view DRR image.

Since a DRF will be clamped on the spinal process or other instruments such as retractors will be used during spinal surgery, their metal properties will produce dark images on the C-Arm images and so influence the robust and accuracy of image similarity measure. Here, we propose to copy the same image of the DRF or instrument into the DRR images so both C-Arm and DRR images will have same noisy images. An example is shown in Figure 7. Figures 7(a) and 7(d) are the AP- and LA-view images, respectively, with the DRF clamper within the C-Arm image areas. The instrument images are segmented by using region growth algorithm as shown in Figures 7(b) and 7(e). The segmented images are added to the C-Arm image areas to generate the masks of Figures 7(c) and 7(f), which are added to the corresponding C-Arm and DRR images as shown in Figure 8 to generate effective images with the same noisy images for accurate image similarity measure.

### 2.4. Experiment of Optimum Registration

To have the optimum registration or transformation matrix between the C-Arm and CT images, optimization method is applied to iteratively estimate the pose (three translations and three rotations) of the CT model so that the image similarity of the DRR and C-Arm images will be best. In this study, three optimization methods are adopted, which are the gradient-based Powell's method, the geometric-based downhill simplex algorithm, and probabilistic-based genetic algorithm [6, 18]. The objective function of optimization is defined as the similarity measure of the C-Arm and DRR images. Six similarity measure methods [14] are proposed, which are Normalized Cross-Correlation (NCC), Gradient Correlation (GC), Pattern Intensity (PI), Gradient Difference Correlation (GD), and Mutual Information (MI). Since C-Arm image-assisted navigation system requires AP- and LA-view images to determine the spatial position of the target, the image similarity measure is defined as the average of the two measures corresponding to AP- and LA-view images.

**Figure 9:**
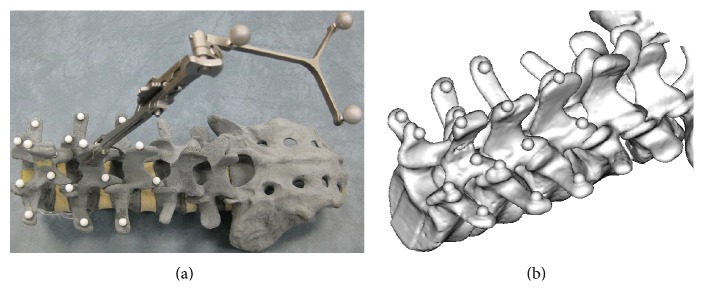
(a) The vertebra phantom with fiducial markers and a DRF attached. (b) The reconstructed CT model.

This experiment aimed to evaluate the registration efficiency and accuracy of the fifteen combinations of the three optimizations approaches with the five similarity measure methods. The vertebra phantom used in the experiment is a saw bone model with spherical fiducial markers attached as shown in Figure 9(a). It was scanned by a Siemens Somatom Sensation 16 Multislice CT with a resolution of 0.46 mm × 0.46 mm × 0.7 mm (pixel size 512 × 512, 400 slices) and shot by a GE OEC 7700 C-Arm with 9′′ image plane as shown in Figure 1.  Figure 9(b) shows its reconstructed CT model. The DRR images were constructed by ray-casting algorithm due to its better image quality. Since the vertebra phantom is deformable, only single body was selected as the ROI for registration. The average time spent on the DRR image construction by using the CUDA accelerator was about 0.01 s.

The spatial coordinates of the fiducial markers are measured by the optic tracker while their image coordinates are detected from the CT images through image process. The transformation matrix between the two coordinate sets can be determined by using interactive closest point (ICP) algorithm, which is the ground truth and is defined as *T*_GT_. Then, the pose estimation of the CT model is down to have optimum image similarity between the C-Arm and DRR images. The transformation matrix of this 2D-3D registration is defined as *T*_2d3d_. The two transformation matrixes are used to define the target registration error (TRE) as

(2) $$\begin{array}{c} \text{TRE}\left(\;P,T_{2\text{d}3\text{d}},T_{\text{GT}}\;\right)=\left\|\;T_{2\text{d}3\text{d}}P_{\text{ct}}-T_{\text{GT}}P_{\text{ct}}\;\right\|, \end{array}$$

where *T*_2d3d_ is the transformation matrix obtained by 2D-3D registration and *P*_ct_ is the CT image coordinate of the fiducial marker.

**Figure 10:**
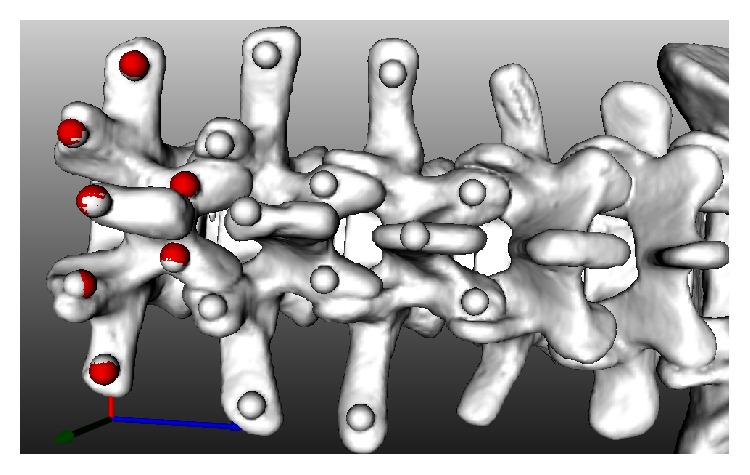
The graphic illustration of the registration result of the seven markers.

The root mean square errors of the ICP registration of seven fiducial markers on a single body are *x* = 0.34 mm, *y* = 0.28 mm, and *z* = 0.26 mm, which is illustrated by Figure 10.

In the beginning of the optimum registration process, three visually identical feature points on the same body were selected from the C-Arm images and CT model, and the initial registration (or transformation matrix) of the C-Arm and CT image frames can be determined by using the coordinates of the three feature points. The purpose is to enable the control of search range of the six translation and rotation parameters (*T*_*x*_, *T*_*y*_, *T*_*z*_, *R*_*x*_, *R*_*y*_, and *R*_*z*_) to be within 5 mm in displacement and 5 degrees in angle relative to the parameters obtained by the initial registration.

## 3. Result

**Figure 11:**
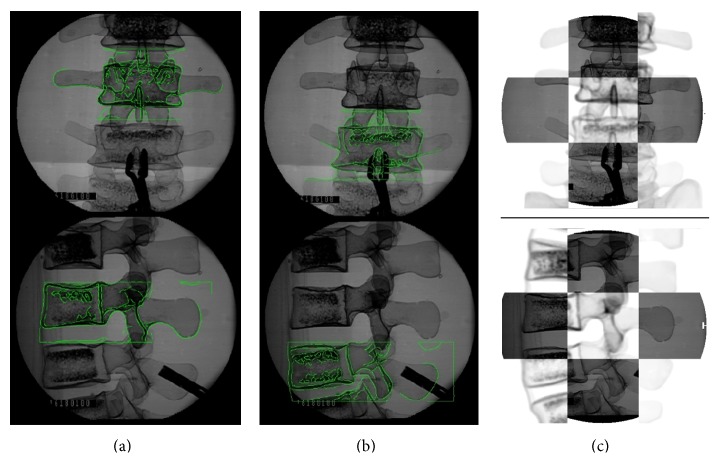
Visual validation of single-body registration without instrument (a) and with instrument (b) and superimposed images (c).

**Figure 12:**
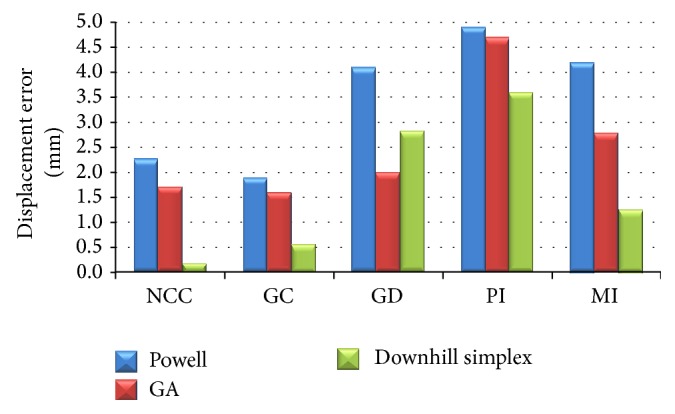
Displacement errors (mm) of fifteen combinations.

**Figure 13:**
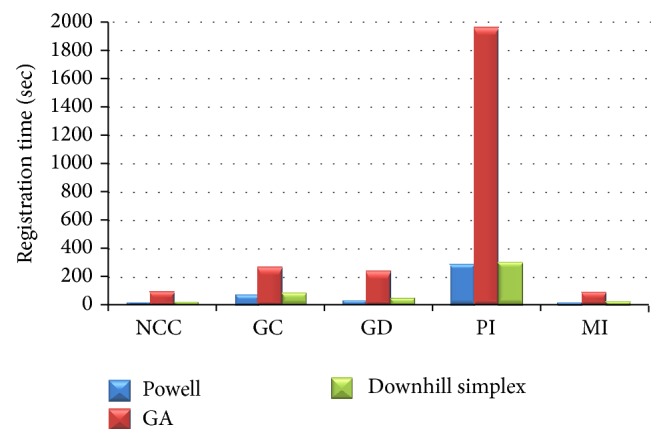
Registration time (sec) of fifteen combinations.

Nine sets of the six initial position and orientation parameters are given randomly for the fifteen combinations of the three optimizations approaches with the five similarity measure methods.  Figure 11 shows an example of registration result by visual validation of the DRR image contour overlapping the original C-Arm image. The displacement errors and registration time are shown in Figures 12 and 13. The performances of the Powell method in displacement error (or registration accuracy) and the genetic algorithm in registration time were poor. The downhill simplex algorithm with the NCC similarity measure method showed that the average displacement error was 0.18 ± 0.02 mm and the average angular error was 0.23 ± 0.05°. Moreover, the displacement errors and angular errors of the NCC with any of the three optimization methods were less than 1 mm and 1° and the registration times were between 10 and 21 seconds. It was observed that the nongradient-based image similarity measuring method NCC had a much better effect in this study, whereas the gradient measuring method GC had a worse effect due to image edge differences and background noise. However, both NCC and GC methods had better performance than the other three methods, because the gray levels of the C-Arm and DRR images were linearly dependent. This image feature conformed to the similarity measure characteristics of NCC and GC, meaning that the linear brightness and contrast variation of the C-Arm and DRR images would not influence the measure result.

In order to find out the adaptation of convergence range of the combination of the downhill simplex optimization approach with the NCC objective function, four convergence intervals are given by (±5 mm, ±5°); (±10 mm, ±10°); (±10 mm, ±15°); (±15 mm, ±10°). For each of the intervals, a total of 40 data sets were sampled randomly.  Table 1 lists the small displacement errors (excluding failure) and large displacement errors (including failure) in the different convergence ranges, so as to select the appropriate interval of convergence. It is obvious that the convergence accuracy and time are positively proportional to the convergence intervals. The larger the convergence interval is, the more the convergence error and time are. For the reasonable convergence interval (±10 mm, ±10°), the average displacement error was 0.22 ± 0.01 mm, the mean convergence time was 16.18 ± 3.6 seconds, and the success rate was 90%.

## 4. Discussion

C-Arm image-assisted surgical navigation system has been broadly applied to orthopedic surgery. For spinal surgery, accurate path planning on the C-Arm AP image is difficult due to lack of the information about axial view of vertebrae that is the key in the placement of pedicle screws. Therefore, the applicability of the C-Arm guided of navigation system is restricted. 2D C-Arm/3D CT image registration is the resolution method to improve the weak point about C-Arm guided of navigation system. A good transformation matrix depends on rapid and effective 2D C-Arm/3D CT image registration method between C-Arm and CT image coordinate frames. Through the transformation matrix, the preplanned surgical path or implant model on preoperative CT images can be transformed and displayed real time on the C-Arm images for surgical guidance. During operation, the locations of surgical instruments will also be displayed on both CT and C-Arm images to help the surgeon to precisely and safely position surgical instruments.

The key in the image-assisted surgical navigation system is to establish an accurate registration relationship between the patient and the before-operation CT images during the operation, in order to implement noninvasive 2D-3D registration. Among the numerous image registration methods, Markelj et al. [6] divided the existing rigid registration methods for 2D images and 3D medical images into three types according to the data volume of the image features: feature-based [8–10], gradient-based [2, 11], and intensity-based [1, 12–14]. In 2D-3D registration, the 2D C-Arm image and the 3D CT image must be consulted in the same coordinate system. There are three registration methods for this, according to the image dimensions and positional connection: (1) the projection algorithm, which converts a 3D image to 2D space via a coordinate system for 2D-2D registration; (2) the back-projection algorithm; and (3) the 3D reconstruction algorithm, which converts a 2D image to 3D space for 3D-3D registration. The similarity is maximized by matching the image contour, image gradient, or image gray scale of the object. The registration result can coordinate the spatial location of corresponding points on two images. The main differences between 2D and 3D registration methods are in the image dimensions and the image features.

2D-3D registration aims to complete an accurate registration process within a short time, in order to improve the practicability in clinical operations. The accuracy of feature-based registration directly depends on the accuracy of segmentation, and it is therefore difficult to perform fully automatically.

Our study compares several methods to find the better calculated methods for 2D-3D registration. We found that the performances of the Powell method in displacement error (or registration accuracy) and the genetic algorithm in registration time were poor. The downhill simplex algorithm with the NCC similarity measure method showed better result. The average displacement error of this method was 0.18 ± 0.02 mm and the average angular error was 0.23 ± 0.05°. Moreover, the displacement errors and angular errors of the NCC with any of the three optimization methods were less than 1 mm and 1° and the registration times were between 10 and 21 seconds. The results of our studies show that the combination of NCC measure method with downhill simplex algorithm obtains maximum correlation and similarity in C-Arm and Digital Reconstructed Radiograph (DRR) images.

## 5. Conclusion

This research studies the registration of 2D C-Arm and 3D CT images for an image-assisted navigation system for spinal surgery. The registration efficiency and accuracy of the fifteen combinations of three optimization approaches with five image similarity measure methods are evaluated. According to the result of our study, this DRR image was rapidly generated by ray-casting algorithm and CUDA parallel program development environment. Among the fifteen combinations for registration, the downhill simplex optimization method with the NCC image similarity measure method had shown the best performance in convergence accuracy and time, which demonstrated the clinic applicability of the combination of 3D CT and 2D C-Arm in image-assisted spinal surgery. The surgical paths can be planned on 3D CT model, transformed into the C-Arm images, and guided by the C-Arm assisted navigation system, which add the spatial information of 3D CT images to the 2D C-Arm images.

## Figures and Tables

**Table 1:** Convergence results of different convergence interval.

Convergence intervals (mm, degree)	(5, 5)	(10, 10)	(10, 15)	(15, 10)

Displacement error (mm)	0.21 ± 0.03	0.22 ± 0.01	0.2 ± 0.01	0.19 ± 0.01

Mean convergence time (sec)	12.9 ± 2.1	16.18 ± 3.6	17 ± 4.6	18.2 ± 4.9

Success rate of convergence	100%	90%	75%	72.5%
